# Reduction in Sulfur Diffusion in Recycled Ground Rubber-Containing Compounds to Improve Tensile Strength †

**DOI:** 10.3390/polym17212942

**Published:** 2025-11-03

**Authors:** Stefan Frosch, Volker Herrmann, Fabian Grunert, Anke Blume

**Affiliations:** 1 Faculty of Plastics Engineering and Surveying, Technical University of Applied Sciences Würzburg-Schweinfurt, 97070 Würzburg, Germany; 2 Elastomer Technology and Engineering (ETE), University of Twente, 7522 NB Enschede, The Netherlands

**Keywords:** rubber, ground rubber, rubber recycling, sulfur diffusion, prevulcanization, crosslink density

## Abstract

Recycling end-of-life rubber to compound components for new formulations is one of the most promising ways to reach the sustainability goals of the rubber industry. Today, devulcanization and pyrolysis are both methods to reuse crosslinked elastomers. A third recycling approach is to process end-of-life rubber into ground rubber (GR), which is then added to green compounds. However, free sulfur diffuses during mixing, storage and vulcanization from the matrix material into the GR particles. As a result, the crosslink density in the matrix is reduced, which deteriorates the in-rubber properties of GR-containing vulcanizates compared to those that do not contain GR. Therefore, GR particles are mainly used today for rubber parts with less demanding dynamic-mechanical requirements, which limits the use of the particles. This study presents an approach for reducing the sulfur diffusion from the matrix into the GR particles by prevulcanizing the green matrix material. This leads to GR-containing vulcanizates with significantly improved mechanical properties. This new approach shows that the quality of the recycled rubber product can be significantly increased by blocking the sulfur diffusion. Even though such prevulcanization is currently only feasible under laboratory conditions, it might also pave the way for finding solutions in a production scale for an effective incorporation of GR into new rubber compounds.

## 1. Introduction

In terms of sustainability and the circular economy, the rubber industry is seeking ways to reduce energy consumption and CO_2_ emissions in the manufacturing of rubber products. Recycling plays a key role in achieving such a reduction. Efforts are being made to further process polymer material from end-of-life tires (ELTs) by pyrolysis [1,2], devulcanization [3,4] or refining into ground rubber (GR) [5,6] as an additive for new products. This recycled additive reduces the absolute need for virgin material of a new vulcanizate and thus contributes to the overall goal of the circular economy. In this study, the recycling method of GR is examined in more detail, and a solution is presented to improve the tensile properties of GR-containing compounds.

To date, shredding and grinding ELTs into small particles is common practice. The size of these particles ranges roughly from diameters from 20 to 40 mm (“chips”), to 1–20 mm (“granulate”), and down to 0–0.4 mm (“powder”) [7]. In this study, GR is referred to as particles with diameters between 0.2 and 2 mm, as previous measurements [8] have shown.

The incorporation of GR particles into green rubber during compounding is an approach for recycling the polymer fraction of ELTs. However, the properties of GR-containing vulcanizates are deteriorated in comparison to those that do not contain the recycled material [9,10,11,12,13,14,15,16]. The main reason for this effect is the diffusion of sulfur from the surrounding green matrix into the GR particles [9,14,15,17,18,19]. This diffusion out of the uncured compound leads to a reduction of the sulfur concentration in the matrix and consequently to a reduction in crosslink density (CLD) within the matrix [19,20]. At the same time, the sulfur concentration increases in the GR particles, which leads to particles with a higher CLD [19,20]. This diffusion effect causes an inhomogeneous vulcanizate in terms of different local sulfur concentrations and thus different local viscoelastic properties [19]. The combination of stiff GR particles in a flexible matrix is therefore responsible for the deteriorated in-rubber behavior of the vulcanizate.

It could be shown in [19] that the concentration difference of free, i.e., not covalently bound soluble sulfur (cyclo-S_8_) between the matrix and GR particles is the driving force of the curative diffusion, as the concentration of free cyclo-S_8_ is high in the green matrix. In contrast, the concentration of free cyclo-S_8_ is low in the GR particles because these are vulcanized, and thus the sulfur is covalently bound. When GR is incorporated into the green matrix compound, free cyclo-S_8_ diffuses from the matrix into the GR particles to reestablish a concentration equilibrium of free cyclo-S_8_ in both phases. The diffusion ends when both phases contain the same amount of free cyclo-S_8_. However, the diffusion also ends with the start of the vulcanization process in both phases. This also explains why the sulfur diffusion from the matrix into the GR particles is even more pronounced when the scorch time of the GR is shorter than that of the surrounding matrix: with a short scorch time of the GR, the sulfur diffuses from the surrounding matrix into the GR until final vulcanization, as the sulfur is only bound in the matrix at a later stage. Thus, the concentration difference of free cyclo-S_8_ between matrix and GR is maintained until the vulcanization also starts in the matrix, which significantly reduces sulfur diffusion.

Phadke et al. [15,18] developed an approach for counteracting the effects of sulfur diffusion. They achieved higher sulfur concentrations in the vulcanized matrix by increasing the amount of both the sulfur and the also mobile accelerator in the green matrix compound before crosslinking. Sulfur diffusion continues to occur, as described above. However, the increased sulfur content in the matrix led to a higher sulfur equilibrium between both the matrix and GR particles. As the matrix became supersaturated with sulfur, it continued to release it through diffusion. However, due to the overall high sulfur concentration, the matrix retained a sufficient amount of sulfur. A satisfactory CLD in the matrix thus was formed and the deterioration of in-rubber properties of the entire vulcanizate is less severe. Until today, this method is a good way to counteract the negative influence due to the incorporation of GR particles [6,19,21].

Therefore, this technique is a first step in reducing the detrimental effects of sulfur diffusion. Nevertheless, sulfur enrichment in the GR continues to reduce the homogeneity between matrix and GR particles with respect to e.g., CLD. Furthermore, the use of a sulfur supersaturated matrix has the disadvantage of allowing diffusion to proceed unimpeded. For example, GR particle solubility and crosslinking kinetics will alter if its compound formulation changes. Thus, for every variation in GR type and quantity, the sulfur concentration may need to be adjusted. Determining the ideal ratio between GR quantity and sulfur supersaturated matrix requires long and laborious preliminary testing [19].

To minimize the deterioration of the properties of GR-containing vulcanizates, it therefore seems more appropriate to prevent sulfur diffusion. Three approaches were investigated in [19]. It could be shown that a sulfur donor system does not lead to reduced sulfur diffusion during vulcanization conditions. In contrast, the use of polymeric sulfur could significantly reduce the diffusion of sulfur during mixing and storage conditions. However, during the vulcanization process, the polymeric sulfur converts back into free cyclo-S_8_ and thus leads to a diffusion behavior similar to that described above.

The third approach, however, showed promising results in reducing sulfur diffusion and thus increasing the in-rubber properties of GR-containing vulcanizates. It was shown that sulfur diffusion mainly occurs during mixing, storage and the scorch time of the curing process due to the elevated temperatures. At the same time, it was demonstrated that sulfur diffusion is mainly limited to the pure form of free cyclo-S_8_. In contrast, sulfur species in combination with ZnO, stearic acid or accelerators are significantly less mobile in the elastomeric matrix. These species can be, e.g., ZnS or the active sulfurating agent complex that are formed during mixing and the scorch time of the crosslinking process [19].

For these reasons, the idea of prevulcanization of the matrix before the incorporation of GR particles was introduced. The current process thus enables the pure cyclo-S_8_ to diffuse from the matrix into the GR particles during mixing, storage and vulcanization processes. In contrast, large proportions of the added free cyclo-S_8_ are first converted into a less mobile sulfur species in the matrix during prevulcanization, as explained above. The reduced mobility of the sulfur-containing species is expected to lead to less sulfur diffusion from the matrix into the subsequently incorporated GR particles. This hypothesis of prevulcanization will be investigated in the following sections.

## 2. Materials and Methods

For the following investigations, the same experimental approach was used as already described in previous publications [19,20,22]. In total, the same compounds were produced five times together with the previous work cited and all vulcanization curves were compared with each other, so that in each case a very good match could be established and reproducibility was ensured.

### 2.1. Process Chain Overview

**Figure 1:**
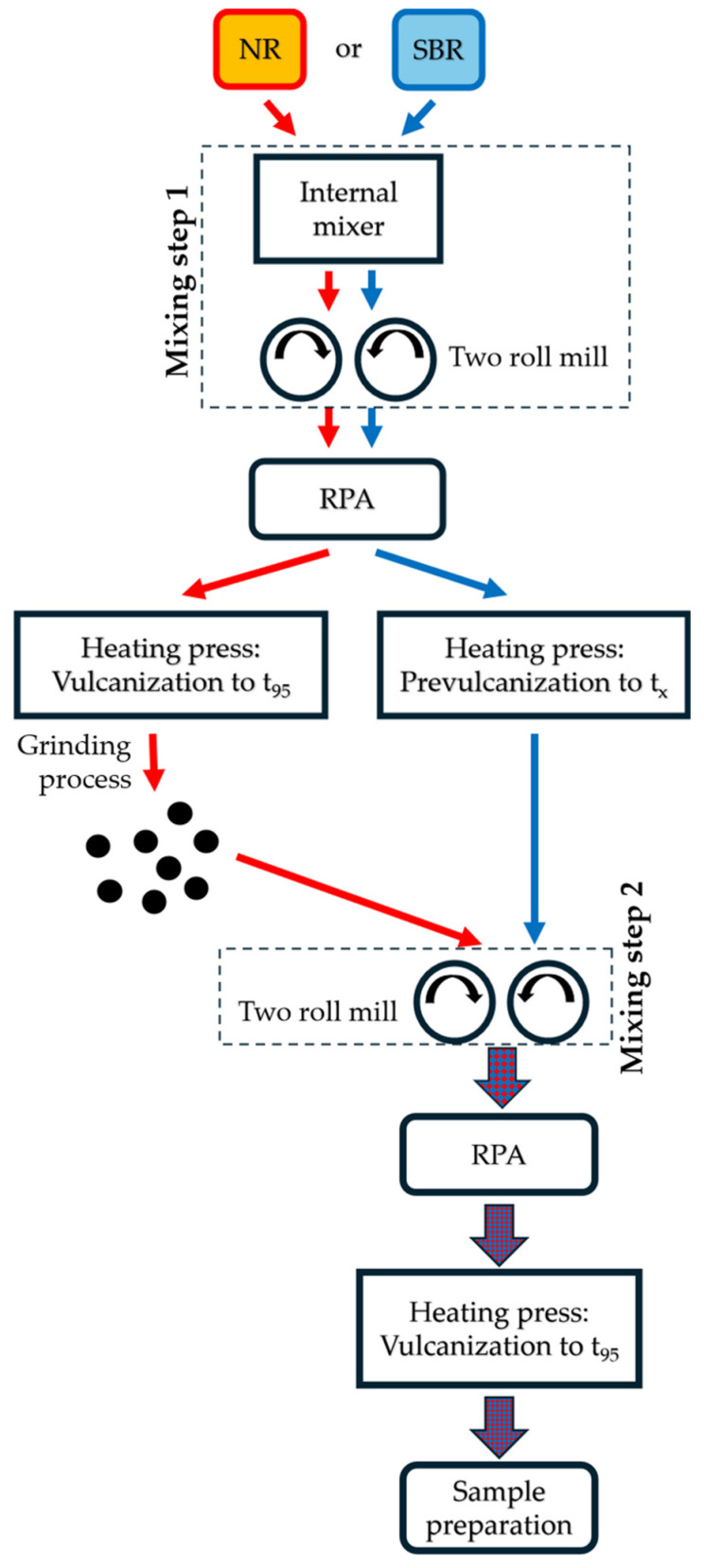
Process chain overview.

The entire process chain, from compounding to the finished test specimens, is illustrated in Figure 1. First, the natural rubber (NR, red) and styrene–butadiene rubber (SBR, blue) compounds are produced separately in mixing step 1. The vulcanization properties at 155 °C of both compounds are then determined in the rubber process analyzer (RPA). With this vulcanization information, the NR compound (red) is vulcanized to t_95_ and formed into 6 mm thick sheets (130 mm × 117 mm). The NR sheets are then converted into GR in a mill, as described in [8]. This material is thus defined as “NR-GR”. Alternatively, the SBR compound (blue) is prevulcanized to the vulcanization time t_x_ with x < 10 in the available heating press to 6 mm thick sheets. This prevulcanization of the matrix compound is the only difference to the current state of the art in processing GR. To date, the GR is incorporated into a non-prevulcanized matrix. The material pairing of NR-GR in an SBR matrix was chosen because [19] showed that sulfur diffusion is pronounced in this material combination, since t_95_ of the NR-GR material is 4 times lower than that of the surrounding SBR matrix material.

In mixing step 2, the NR-GR is incorporated into the prevulcanized SBR matrix on a two-roll mill. The crosslinking characteristics of the new compound (red-and-blue-colored arrows) are then determined again in the RPA. With this information, rubber sheets with a thickness of 2 and 6 mm are vulcanized to t_95_. These sheets are converted into test specimens for the following examinations.

The following sections describe the separate production steps and the equipment used in more detail.

### 2.2. Compound Formulations and Mixing Conditions

This study investigates the diffusion behavior of soluble sulfur between the SBR matrix and the NR-GR material. The formulations, as well as the names and manufacturing companies of the individual compound components, are depicted in Table 1. The NR vulcanizate was manufactured to be subsequently converted into GR and then to be incorporated into compounds of SBR. In total, three SBR compounds were manufactured, each prevulcanized to different t_x_. Details are explained in the following table.

The compound components described in Table 1 were mixed in mixing step 1 using an internal mixer (Werner & Pfleiderer, 1.5 l, PES3) and a laboratory mill (Schwabenthan 200 × 450). Mixing step 1 is a two-stage mixing process, divided into the preparation of the base and the final compounds. An overview of the mixing sequence and the key parameters of the base compounds can be found in Table 2. Prior to the compounding of the base compounds, 1 kg of the NR is masticated in the internal mixer for 20 min at 20 rpm and 25 °C.

Table 3 depicts manufacturing details of the final compounds of mixing step 1.

The NR vulcanizate was then ground in a centrifugal mill (see Section 2.4). In contrast, the SBR compounds were prevulcanized to t_x_ and formed into 6 mm thick sheets with dimensions of 130 mm × 117 mm. Each sheet was then cut with common rubber scissors into four stripes to increase the processability on the two-roll mill in mixing step 2.

In the latter compounding process, 30 phr of NR-GR was incorporated on the two-roll mill into the prevulcanized SBR matrix (see mixing step 2 in Figure 1). This compound is referred to as “SBR&NR-GR” in the remainder of this article. Table 4 describes the compound constituents of SBR&NR-GR in detail.

The procedure of incorporating 30 phr NR-GR into a prevulcanized SBR-matrix in mixing step 2 is displayed in Table 5.

### 2.3. Vulcanization Behavior

Subsequent to mixing steps 1 and 2, specimens were cut from the compound sheets with the SIS-VS punch press from TA Instruments (New Caste, Delaware, USA, former Scarabaeus) for the following investigations with the RPA. The latter is a SIS V50 from TA Instruments (former Scarabaeus) and was used to determine the curing behavior of all samples at 155 °C, with a frequency of 1.667 Hz and an oscillation angle amplitude of 0.50° within a measurement time of 60 min. Every compound was measured three times. The rheometer was equipped with the software SQS—RPA Monitor Version 6.15.

### 2.4. Mill

A centrifugal mill, ZM200, from the company Retsch (Haan, Germany), with a 12 tooth rotor and a sieve with a mesh size of 2 mm was used to grind the NR at a frequency of 18.000 rpm. To prepare the samples for this grinding process, the 6 mm thick sheets of NR were cut with common rubber scissors into approximately 6 × 6 × 6 mm^3^ cubes. Subsequently, these cubes were cooled below their glass transition temperature (Tg) using liquid nitrogen. Then, they were placed in portions of several 10 g into the mill. In the centrifugal mill, the vulcanizate cubes were further reduced to GR. The SBR material was not ground.

### 2.5. Heating Press

The heating press Polystat 200 T from Servitec (Wustermark, Germany) was used to prevulcanize the SBR matrix to t_x_ and to vulcanize both the NR and SBR&NR-GR to t_95_. The heating press was equipped with two molds to produce both 2 and 6 mm thick sheets with dimensions of 130 mm × 117 mm.

### 2.6. Micro X-Ray Fluorescence Analysis

A micro X-ray fluorescence analysis (μ-XRF) was employed to investigate areas of 10 mm × 10 mm of the vulcanized 6 mm thick sheets. This technique is used to qualify and quantify chemical elements on (rubber) surfaces in an imaging manner [22,23,24,25]. For the investigations, an M4 Tornado from Bruker Nano Analytics (Berlin, Germany) was equipped with a rhodium tube that was set to 200 μA and 50 kV. The target material was rhodium, no filter was chosen. The diameter of the X-ray beam was set to 25 μm, identical to the resolution (distance between two measurement spots). Each spot was measured three times for 40 ms. The fluorescence radiation was detected with two silicon drift detectors. To enhance the sulfur signal by eliminating the argon present in the ambient air, the sample chamber was evacuated to an absolute pressure of 20 mbar for the analysis. This was necessary, since the fluorescence energies of both elements are similar [26]. The software Esprit M4 (version: 1.6.0.286) was used to qualify and quantify the local sulfur concentrations [19].

### 2.7. Tensile Tests

S2 tensile test specimens were cut out of the 2 mm thick vulcanized rubber sheets using a Zwick punch press 7103 (Ulm, Germany). These specimens were then investigated using a Zwick 1474 tensile testing machine (Ulm, Germany) based on DIN 53,504 with a crosshead speed of 50 mm min^−1^. The software Doli Test & Motion, version 4.6.0.6, was used to process the data.

### 2.8. Measurement Errors

For all results, mean values were calculated, which are listed in the following chapter. These mean values are presented with their measurement error, calculated using the Student’s t-test with a 95% confidence interval. This means that the mean values reported in this article, including their measurement errors, are valid with a 95% probability [27,28].

## 3. Results

The results of the different test methods are described in the following section.

### 3.1. Vulcanization Behavior

**Figure 2:**
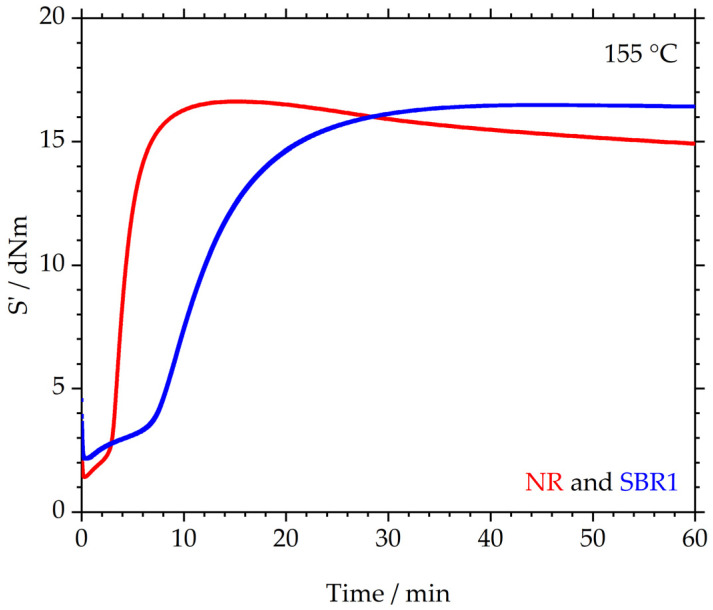
Crosslinking behavior of NR and SBR1: median curves (maximum of S’) of three measurements each.

The crosslinking behavior is depicted in Figure 2. The elastic torque S’ in dNm is plotted over the measurement time in min. The median curves for S’_max_ from three measurements of the NR and SBR1 (one of three investigated SBR compounds) are illustrated. The NR shows a relatively short scorch time, after which S’ rises to a maximum value of 16.6 ± 0.2 dNm. In the following, S’ decreases again, indicating that the material shows reversion. Compound SBR1, however, behaves differently: the material reaches a plateau with a value of 16.5 ± 0.4 dNm for S’; therefore, the maximum S’ values of NR and SBR1 are at an identical level, taking the measurement error into account. Furthermore, the scorch time of SBR1 is significantly longer than that of NR, although identical concentrations of sulfur and accelerator were used in both compounds. This increased scorch time could be due to the retarding effect of the added ZnO in the SBR, as described in [29]. In this article, it was found that the postulated vulcanization process described by Morrison and Porter [30] applies to NR. In contrast, it was found that for other polymers, such as high vinyl SBR, other ring opening and vulcanization reactions occur, and that ZnO can exhibit a retarding effect in these processes. For example, sulfur can accumulate in the ZnO crystal and can therefore not participate in vulcanization reactions [31].

In Table 6, characteristic values of the RPA measurements of all analyzed samples are listed. Each compound was measured three times, with the mean values including errors for t_10_, t_50_, t_95_ and S’_max_ given in each case. It is evident that the NR compound cures significantly faster than the three compounds made of SBR (SBR1, SBR2 and SBR3).

The three SBR compounds each have a t_10_ of approximately 7 min. Preliminary tests were carried out, which showed that GR could not be incorporated into t_10_ prevulcanized matrix compounds. This can be explained by the lack of viscous processing behavior of the compound because first crosslinking reactions initiated the transformation of the matrix into a vulcanizate. Accordingly, the prevulcanization time was shortened to enable the incorporation of the recycled material. Consequently, SBR1, SBR2 and SBR3 were prevulcanized to different t_x_ with x < 10. Prevulcanization times of 2, 4 and 6 min (t_2_, t_5_ and t_7_ accordingly) were selected for SBR1, SBR2 and SBR3, before 30 phr of NR-GR was incorporated into each. The GR-containing and prevulcanized compounds are referred to as “SBR1_2min&NR-GR”, “SBR2_4min&NR-GR” and “SBR3_6min&NR-GR”.

**Figure 3:**
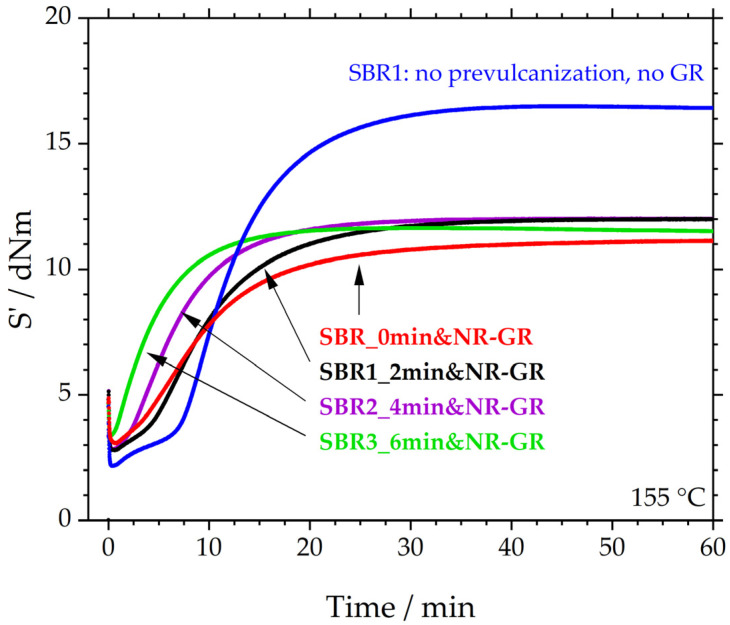
Crosslinking behavior of the prevulcanized and the 30 phr NR-GR-containing compounds SBR1_2min&NR-GR, SBR2_4min&NR-GR and SBR3_6min&NR-GR with the reference compounds SBR1 (compound that was not prevulcanized and does not contain GR) and SBR_0min&NR-GR (not prevulcanized but contains 30 phr NR-GR). Median curves (maximum of S’) were derived from three measurements each.

Figure 3 depicts the median (S’_max_) vulcanization curves of these three compounds. The S’-values of all three GR-containing samples settle on a plateau of approximately 12 dNm. These plateaus are approx. 4 dNm below that of SBR1, which was not prevulcanized, does not contain GR and serves as a reference here. Because the three prevulcanized specimens reach the same plateau, it appears that the degree of prevulcanization has no influence on the resulting S’_max_. Thus, the 4 dNm difference between SBR1 and the three prevulcanized samples corresponds to the general influence of the GR. This influence is underlined by “SBR_0min&NR-GR”, a sample with an identical formulation to the first three specimens. The difference from the other three samples is that the matrix was not prevulcanized before the incorporation of 30 phr of NR-GR. It is therefore a sample that represents the current manufacturing process in the rubber industry. The sample has already been examined in more detail in [12,19]; however, the results are illustrated here again because, together with those of the three prevulcanized specimens, they demonstrate the influence of prevulcanization. It is therefore evident that the reduction of S’_max_ is an effect of the addition of GR and not of prevulcanization (see also Table 6).

In comparison, however, the scorch time decreases significantly with the degree of prevulcanization. The reactions of all three prevulcanized samples start earlier than the SBR1 reference. The scorch time is reduced with increasing prevulcanization: t_10_ decreases from 6.9 ± 0.6 min for SBR1, to 3.9 ± 0.1 min for SBR1_2min&NR-GR, 2.5 ± 0.1 min for SBR2_4min&NR-GR and 1.2 ± 0.0 min for SBR3_6min&NR-GR. This reduction in scorch time can be explained by the matrix material having already been prevulcanized, thereby initiating and interrupting scorch reactions. This scorch status is maintained during the incorporation of NR-GR and then continues at crosslinking temperatures.

The non-prevulcanized reference SBR_0min&NR-GR also exhibits a shorter scorch time compared to SBR1. This is consistent with the findings of Gibala and Hamed [14] and can be explained by the diffusion processes of accelerator fragments from the GR particles to the matrix material, speeding up vulcanization.

The comparison with SBR1 (blue) reveals that S’_min_ is higher for the other four samples. This is because each of them contains 30 phr of NR-GR, i.e., already crosslinked material, which increases the initial torque. It becomes apparent that the 6 min prevulcanized specimen (green) exhibits the highest initial torque. The possible reason for this could be that parts of the matrix had already crosslinked slightly, increasing S’_min_.

**Figure 4:**
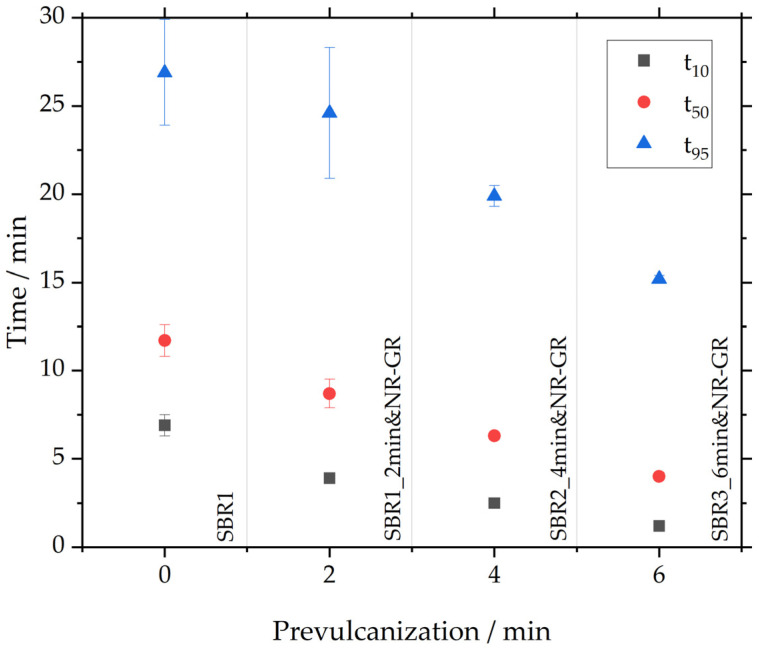
The influence of prevulcanization on t_10_, t_50_ and t_95_.

The exact t_10_, t_50_ and t_95_ can be found in Table 6 and in Figure 4. The latter shows t_10_, t_50_ and t_95_ of the investigated samples from Figure 3 during the prevulcanization of the matrix material. It is evident that all three characteristic times decrease linearly as prevulcanization progresses. This reduction can be explained by the matrix material having already been prevulcanized, thereby both initiating and interrupting scorch reactions. Longer prevulcanization times of the matrix compound thus lead to shorter characteristic vulcanization times of the NR-GR-containing compounds.

### 3.2. Micro X-Ray Fluorescence Analysis

**Figure 5:**
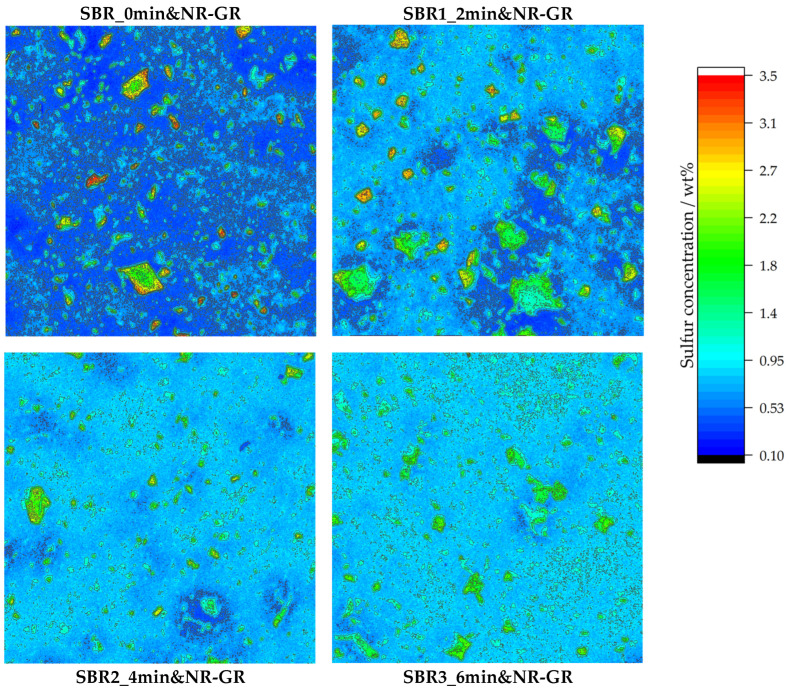
Local sulfur concentrations detected via µ-XRF on different vulcanizate surfaces with measurement areas of 10 mm × 10 mm for every specimen. The GR particles are identifiable by their higher sulfur concentration compared to the surrounding matrix. With increasing prevulcanization of the matrix material, the homogeneity of the sulfur distribution increases. Levels of matrix prevulcanization: 0, 2, 4 and 6 min (from top left to bottom right).

Using µ-XRF, the local sulfur concentrations of the three samples with different degrees of prevulcanization SBR1_2min&NR-GR, SBR2_4min&NR-GR and SBR3_6min&NR-GR, as well as those of the non-prevulcanized reference SBR_0min&NR-GR, were determined, investigating areas of 10 mm × 10 mm. The results can be found in Figure 5.

The non-prevulcanized sample SBR_0min&NR-GR—top left in Figure 5—shows an inhomogeneous sulfur distribution because of the diffusion processes during the mixing, storage and crosslinking steps [19]. The GR particles can be identified by their green-to-red coloration. The local sulfur concentration is therefore approx. 1.8–3.5 wt%. Considering that the initial concentration according to the formulation was only 0.93 wt%, it can be concluded that the sulfur concentration in the GR particles is locally doubled to quadrupled. The reason for the sulfur enrichment is that the latter diffuses from the matrix into the GR particles due to the concentration differences in free sulfur between the matrix and GR [19]. This depletes the matrix of sulfur, which can be distinguished from the GR particles by its dark blue coloration. A sulfur concentration of approx. 0.4 wt% is achieved within the matrix, i.e., approximately halving the sulfur concentration according to the initial formulation (0.93 wt%). In [19], it is shown that the local viscoelastic properties also change with the local inhomogeneous sulfur distribution, whereby the in-rubber properties of the entire vulcanizate are deteriorated.

Sample SBR1_2min&NR-GR (top right in Figure 5) also shows an inhomogeneous local sulfur distribution. Higher sulfur concentrations are found in the GR particles compared to in the surrounding matrix. Compared to sample SBR_0min&NR-GR, however, it is evident that there are fewer red zones with sulfur concentrations of 3.5 wt% in the GR particles. At the same time, more GR particles are colored green, which indicates a local sulfur concentration of approx. 1.8 wt%. It is therefore clear that less sulfur accumulates in the GR particles if the matrix was prevulcanized for 2 min before incorporating the NR-GR. There are still regions in the matrix with a concentration of approximately 0.4 wt% because 2 min of prevulcanization could not fully stop the sulfur diffusion from the matrix into the GR. However, there are also areas in the matrix which, at around 0.9 wt%, are within the target range according to the compound formulation. It is therefore evident that a matrix prevulcanization of 2 min already restricts sulfur diffusion.

If the prevulcanization is extended to 4 min—bottom left in Figure 5—it reveals that sulfur diffusion is further restricted. Most GR particles are displayed in green, which corresponds to a local sulfur concentration of approx. 1.8 wt%, and thus a doubling of the initial concentration (0.93 wt%) and at the same time a halving in comparison to the concentrations of the non-prevulcanized sample (3.5 wt%). The extremely low-sulfur zones in the matrix become steadily smaller. Most of the matrix has a sulfur content of approx. 0.75 wt%, which is closer to the target range (0.93 wt%) according to the compound formulation. Accordingly, sulfur diffusion seems to be further restricted with prolonged prevulcanization.

This trend continues with 6 min of prevulcanization (see bottom right in Figure 5). This specimen exhibits the most uniform sulfur homogeneity of all four analyzed samples. At the same time, an increased sulfur concentration is still determined in the GR particles. However, the difference from the concentration in the matrix is the lowest in this compound in comparison to all other compounds. An extension of the prevulcanization time therefore leads to a more homogeneous distribution of sulfur within the sample.

**Figure 6:**
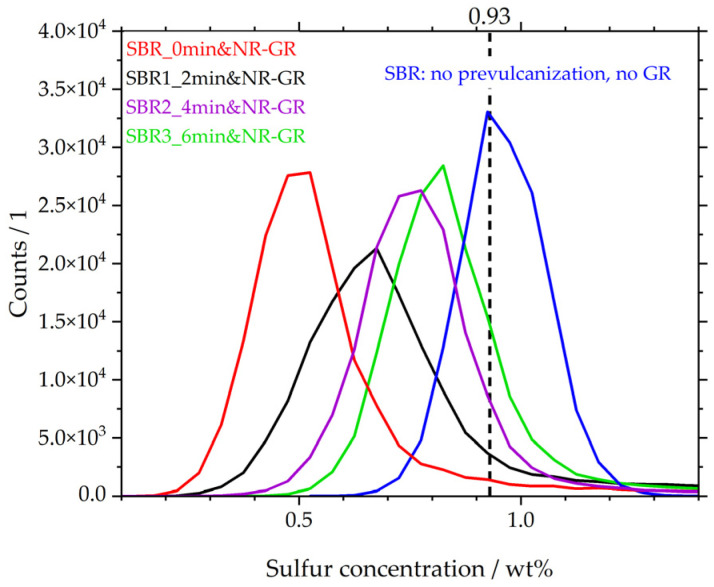
Distribution curves of local sulfur concentrations in the matrices of different vulcanizates.

A summary of the µ-XRF investigations of the local sulfur concentration is depicted in Figure 6 in the form of distribution curves. The locally measured sulfur concentrations are plotted on the abscissa. According to the formulation, both compounds—i.e., NR and SBR—contain 0.93 wt% sulfur. This concentration is plotted on the abscissa as a dashed line. The distribution curve of the sulfur concentration was determined using the matrix material SBR, which has not yet been prevulcanized and does not yet contain NR-GR, shown here as an example (blue curve). The data originates from [19]. The most frequently detected sulfur concentration in SBR is 0.9 wt%, but there are also zones with lower and higher local concentrations of sulfur. The curve of sample SBR_0min&NR-GR (red curve, also originating from [19]) shifts towards lower sulfur concentrations. This means that the matrix has a significantly lower sulfur concentration due to diffusion processes.

However, if the matrix is prevulcanized for 2 min (black curve) before NR-GR is incorporated, the local sulfur concentration changes. The distribution curve shifts to higher concentrations compared to that for the non-prevulcanized sample (red). More sulfur remains in the matrix because of the reduced diffusion behavior of the sulfur species formed during prevulcanization. This behavior increases with longer prevulcanization times of 4 min (purple) and 6 min (green). Compared to the non-prevulcanized sample (red), with a maximum of approx. 0.45 wt%, the vulcanizate with a matrix prevulcanized for 6 min reaches a peak at 0.8 wt%. This behavior supports the hypothesis proposed by [19], that species formed during crosslinking—e.g., the active sulfurating agent postulated by Morrison and Porter [30]—are less mobile than the free sulfur initially present. It is thus evident that with increasing prevulcanization of the matrix, less free sulfur is able to diffuse into the GR particles. As a result, more sulfur remains in the matrix, and the distribution curves shift with increasing prevulcanization to the SBR-reference (blue). Even during the relatively short prevulcanization of 2 min, the content of the less mobile species formed is so high that the distribution curve (black) differs significantly from that of the non-prevulcanized curve (red).

The distribution curves indicate that, apart from the matrix peak, there is no other peak representing the sulfur concentration in the GR particles. Instead, there are higher sulfur concentrations at moderate counts to the right of the matrix peaks. It therefore indicates that the sulfur concentration in the GR particles is less uniform and instead extends over a wide concentration range, e.g., from concentrations of approx. ≥1 wt% in the 2 min prevulcanized sample (black).

Compared to the SBR reference (blue), the matrices of the prevulcanized compounds are comparatively less sulfur-depleted, which leads to the expectation of increased mechanical properties compared to the non-prevulcanized specimen (red), for example. This is investigated in the following section.

### 3.3. Tensile Tests

To assess the influence of prevulcanization on mechanical properties, tensile tests were carried out.

**Figure 7:**
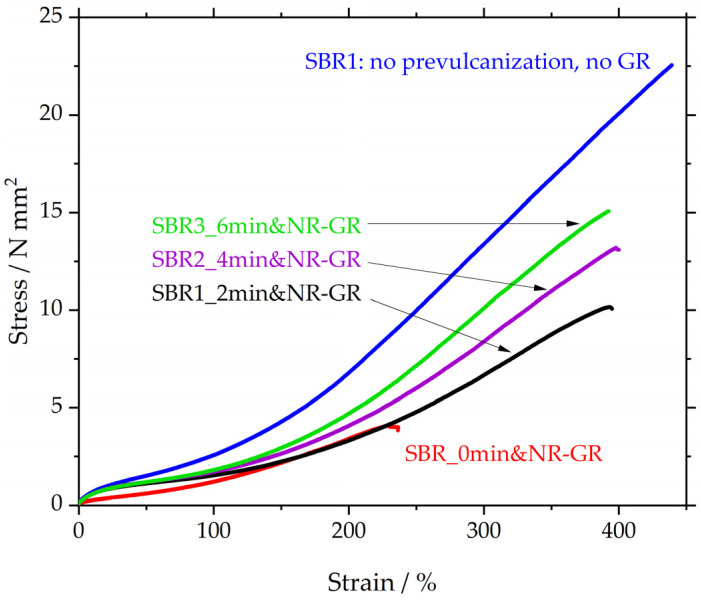
Stress strain curves of SBR1 and vulcanizates containing ground rubber with different degrees of prevulcanization.

In Figure 7 and Table 7, the sample SBR1 (blue) reaches a tensile strength of approx. 22.5 MPa at an elongation at break of 435%. For the same matrix material to which 30 phr NR-GR was added, and for which the matrix was not prevulcanized (SBR_0min&NR-GR, red), the tensile strength and elongation at break are significantly reduced. SBR_0min&NR-GR is again derived from [12,19] and serves as a reference to assess the influence of the prevulcanization.

This influence of prevulcanization is demonstrated by the curves of SBR1_2min&NR-GR, SBR2_4min&NR-GR and SBR3_6min&NR-GR. The samples differ from SBR_0min&NR-GR (red) only in terms of the prevulcanization of the matrix for 2, 4 or 6 min, respectively. A 2 min prevulcanization already leads to a more than doubled tensile strength of 10 MPa compared to the red reference curve, exhibiting only 4.8 MPa. The elongation at break increases from 240% to almost 400%. This significant effect is the result of the prevulcanization step prior the incorporation of GR particles. Extending the prevulcanization time leads to a further increase in tensile strength with the same elongation at break. The significant increase in tensile strength and elongation at break can be explained by the higher sulfur concentration in the matrix and therefore an assumed higher CLD. The fact that the elongation at break for all three prevulcanized specimens remains identical at just under 400% could be due to the GR particles increasing the intrinsic elongation of the surrounding matrix. This leads to a local rise in strain in the vicinity of the particles, leading to failure of the entire specimen when this strain exceeds the elongation at break of the matrix material. The tensile tester, however, detected only one non-local strain value of the entire specimen, which is listed in Table 7 to be between 372 and 395%. This value might not be sensitive enough to distinguish differences between the different prevulcanized samples.

## 4. Discussion

The rubber industry is making efforts to drive towards a more circular economy to increase its sustainability. The recycling of rubber components such as end-of-life tires (ELTs) is an essential part of this procedure. Recycling rubber is challenging due to the covalent crosslinks between the polymer chains formed during the vulcanization process. These bonds define the viscoelastic properties of the vulcanizate. One recycling method involves grinding the ELTs into ground rubber (GR) particles. These particles can then be incorporated into green compounds to reduce the need for raw material. However, GR-containing compounds exhibit significantly deteriorated properties compared to those without GR. This is firstly because the GR particles perform as an inactive filler, increasing the intrinsic elongation of the matrix material in their vicinity. Secondly, free sulfur diffuses from the matrix into the GR particles during compounding, storage and vulcanization, resulting in matrix systems with lower and GR particles with higher crosslink density (CLD).

To improve the properties of GR-containing vulcanizates, the influence of matrix prevulcanization upon the diffusion behavior of sulfur was investigated in this article. It was found that the mobility of sulfur is significantly reduced when it forms different intermediates or species during the vulcanization process. Therefore, it was investigated whether the incorporation of GR particles into a prevulcanized matrix leads to less sulfur diffusion and thus increased tensile properties compared to a compound with the particles being incorporated into a non-prevulcanized matrix.

RPA measurements showed that prevulcanization reduces the scorch time of GR-containing compounds, because the heating phase continues the already started but interrupted vulcanization process during prevulcanization. An influence of the prevulcanization upon the maximum torque could not be detected.

It was demonstrated, by using micro X-ray fluorescence analysis (µ-XRF), that sulfur diffusion significantly decreases with the degree of prevulcanization. This indicates that the mobility of sulfur is reduced in prevulcanized matrix compounds in comparison to those that have not been prevulcanized. In contrast to the conventional production process without prevulcanization, the sulfur content in the matrix could almost be doubled, because less sulfur diffused out of the matrix into the GR particles.

The increased sulfur concentration in the matrix results in the formation of a higher CLD, which has a positive effect both on tensile strength and elongation at break. It was thus possible to increase tensile strength from about 4 MPa to 15 MPa, improving the elongation at break at the same time from about 230% to almost 400%. Besides that, the elongation at break reaches a maximum, indicating the influence of the GR particles in form of an inactive filler.

Prevulcanization is a comparatively simple method on a laboratory scale because it does not need any additional machinery or changes of compound formulations besides the prevulcanization step itself. However, the prevulcanization will face challenges bringing it into industrial scale production because of higher resulting viscosities (compounding, energy consumption) and vulcanization processes (scorching). If the scorching behavior could be tailored with respect to torque rise over a relatively long time, the prevulcanization could be installed into the rubber production procedure. Therefore, prevulcanization might pave the way for effective incorporation of GR into new rubber compounds, even at production scale.

## Figures and Tables

**Table 1:** Compound formulations; all values are given in parts per hundred rubber (phr), adapted from [20].

	NR	SBR	Name	Company
NR	100	-	SIR 20	Weber & Schaer GmbH & Co. KG, Hamburg, Germany
E-SBR	-	100	Buna SE 1502 L	Arlanxeo Deutschland GmbH, Cologne, Germany
N330	50	50	Corax N330	Orion Engineered Carbons S.A., Eschborn, Germany
ZnO	5	5	Zinkoxid Rotsiegel	L. Brüggemann GmbH & Co. KG, Heilbronn, Germany
Stearic acid	3	3	Palmera B1804	KLK Emmerich GmbH, Emmerich am Rhein, Germany
Soluble sulfur	1.2	1.2	K46859483 542	Merck Chemicals GmbH, Darmstadt, Germany
CBS	1.2	1.2	Vulkacit CZ/EG-C	Lanxess Deutschland GmbH, Krefeld, Germany

**Table 2:** Production procedure of base compounds of mixing step 1 [19].

Time/Min	Processing Step of Base Compounds
0	Polymer
1	ZnO, stearic acid, carbon black
2.5	Cleaning step
4.5	Dump
	Weight check
	Sheeted off on laboratory mill (both rollers 20 rpm, gap 2.5 mm, 40 °C) for 1 min. Relaxation for 1 h.

**Table 3:** Production procedure of final compounds of mixing step 1 [19].

Time/Min	Processing Step of Final Compounds
0	Base compound, sulfur, accelerator
3	Dump
	Weight check
	Sheeted off on laboratory mill (front roller 16 rpm, back roller 20 rpm gap 2.0 mm, 40 °C)
0	Final compound on laboratory mill
1	Cutting three times left and right, roll up and blend back at a gap of 1 mm
4	Gap: 2.5 mm, both rollers at 20 rpm, dump

**Table 4:** Compound formulation for mixing step 2: addition of 30 phr NR-GR into a prevulcanized SBR-matrix; all values are given in phr.

	SBR&NR-GR	
E-SBR	100	Prevulcanized to t_x_ with x < 10; cut into 6 mm thick stripes
N330	50	
ZnO	5	
Stearic acid	3	
Soluble sulfur	1.2	
CBS	1.2	
NR-GR	30	

**Table 5:** Production procedure for mixing step 2.

Time/Min	Processing Step of SBR&NR-GR
	Laboratory mill (both rollers 20 rpm, gap 6 mm, 40 °C)
0	Start feeding stripes of the prevulcanized SBR compound onto both rollers
5	All stripes are incorporated; reduction of gap to 2.5 mm
6	Friction: front roller at 16 rpm, back roller at 20 rpm
7	Reduction of gap to 2.0 mm
8	Friction: front roller to 20 rpm, back roller to 16 rpm
9	Cutting two times left and right
10	Adding 30 phr ground rubber
14	3 x roll up and blend back at gap of 1.0 mm
15	Both rollers at 20 rpm, gap of 2.0 mm, dump

**Table 6:** Crosslink characteristics of the investigated compounds.

Compound	t_10_/min	t_50_/min	t_95_/min	S’_max_/dNm
NR	3.0 ± 0.0	4.2 ± 0.2	8.5 ± 0.2	16.6 ± 0.2
SBR1	6.9 ± 0.6	11.7 ± 0.9	26.9 ± 3.0	16.5 ± 0.4
SBR2	7.2 ± 0.3	12.1 ± 0.5	29.2 ± 2.1	16.9 ± 0.3
SBR3	6.8 ± 0.3	11.4 ± 0.8	26.6 ± 2.9	17.1 ± 0.6
SBR_0min&NR-GR *	3.1 ± 0.1	8.6 ± 0.1	28.8 ± 1.3	11.3 ± 0.8
SBR1_2min&NR-GR	3.9 ± 0.1	8.7 ± 0.8	24.6 ± 3.7	12.0 ± 0.3
SBR2_4min&NR-GR	2.5 ± 0.1	6.3 ± 0.2	19.9 ± 0.6	12.0 ± 0.3
SBR3_6min&NR-GR	1.2 ± 0.0	4.0 ± 0.2	15.2 ± 1.6	11.6 ± 0.2

* Reference sample without prevulcanization but identical compound formulation; adapted from [12,32].

**Table 7:** Summary of tensile properties of the investigated vulcanizates.

Specimen	Number of Valid Tests	Mean Stress /N mm^2^ Incl. 95% Error	Mean Strain /% Incl. 95% Error
SBR1	12	21.8 ± 1.0	421 ± 13
SBR_0min&NR-GR	10	4.0 ± 0.3	232 ± 12
SBR1_2min&NR-GR	12	10.1 ± 0.8	379 ± 19
SBR2_4min&NR-GR	11	12.9 ± 0.8	395 ± 17
SBR3_6min&NR-GR	11	15.0 ± 0.5	372 ± 11

## Data Availability

Dataset available on request from the authors.

## Abbreviations

The following abbreviations are used in this manuscript:

µ-XRF	Micro X-ray fluorescence analysis
CLD	Crosslink density
cyclo-S_8_	Soluble sulfur
ELTs	End-of-life tires
GR	Ground rubber
RPA	Rubber process analyzer
S’	Elastic torque
S’_max_	Maximum elastic torque
S’_min_	Minimum elastic torque
t_10_	Time to reach 10% of full vulcanization
t_50_	Time to reach 50% of full vulcanization
t_95_	Time to reach 95% of full vulcanization
Tg	Glass transition temperature
t_x_	Time to reach x % of full vulcanization
